# Determinants of sexual dysfunction in women recovered from COVID-19

**DOI:** 10.1192/j.eurpsy.2022.498

**Published:** 2022-09-01

**Authors:** M. Lagha, G. Hamdi, N. Dhaouadi, S. Chebli, R. Ridha

**Affiliations:** 1 Razi Hospital, Forensic Psychiatry, La Manouba, Tunisia; 2 Abderrahmen Mami Hospital, Preventive Medicine, Ariana, Tunisia

**Keywords:** dysfunction, women, sexuality, Covid-19

## Abstract

**Introduction:**

While several studies have assessed the impact of the COVID-19 pandemic on sexuality and sexual behavior in the general population, very few studies have assessed sexuality in patients recovered from Sars-Cov 2 infection.

**Objectives:**

The objectives of our study were to assess factors associated with sexuality dysfunction in women recovered from covid-19.

**Methods:**

This is a case-control study.

The women in the case group have been infected with Sars-Cov 2, and cured for one to two months at the time of the study, women in the control group have not been infected with Sars-Cov 2. We assessed depression, anxiety, post-traumatic stress disorder (PTSD) and sexuality in both groups using the Beck Depression Inventory (BDI), the Coronavirus Anxiety Scale (CAS), the Post traumatic stress disorder Checklist Scale (PCLS) and the Female Sexual Function Index (FSFI).

**Results:**

In total, we recruited 30 women in the case group and 30 women in the control group. An FSFI score <26.55 and corresponding to impaired sexual function was found in 63.33% of women in the case group versus 53.33% of women in the control group (p=0.009). Factors influencing sexual activity were depression (OR = 17.86, CI95% = [1.1-290.12]) and PTSD (OR = 18.51, CI95% = [1.43-240.30]).

**Conclusions:**

Depression and PTSD are significantly associated with sexual dysfunction in women recovered from COVID-19, even in mild or pauci-symptomatic clinical forms.

**Disclosure:**

No significant relationships.

